# Hoping and waiting for rescue: Concepts, scale development and process

**DOI:** 10.1111/papt.12588

**Published:** 2025-03-28

**Authors:** Paul Gilbert, Jaskaran Basran, Ptarmigan Plowright, Kelly Morter, Malcolm B. Schofield, Jean Gilbert

**Affiliations:** ^1^ Centre for Compassion Research and Training, College of Health, Psychology and Social Care University of Derby Derby UK; ^2^ The Compassionate Mind Foundation Derby UK

**Keywords:** attachment, emotion regulation, helplessness, psychometric, psychotherapy, rescue, self‐help, self‐reliance, social safeness

## Abstract

**Background:**

It is clinically recognised that some people find it difficult to engage with, or commit to, self‐help for life difficulties. This may be due to various reasons such as experiences of helplessness, feeling overwhelmed and lacking skills, and low confidence in the process. Another reason can be beliefs of ‘needing others’ to bring change about; that they are not able to do it for themselves and are ‘hoping and waiting’ for others to ‘rescue’ them.

**Objectives:**

This study developed a new self‐report scale to explore people's experiences of hoping and waiting to be rescued from distressing mental states. Second, we sought to explore how this orientation links to mental health, social relating, early life experiences, and emotion dysregulation.

**Methods:**

The scale comprised 18 items derived from clinical experiences and was completed online by two general population samples from the United Kingdom (total *n* = 445). Participants also completed measures of emotion dysregulation, reassurance‐seeking, depression, anxiety, stress, self‐other relating, social comparison, social safeness, early memories of warmth and parental bonding.

**Results:**

Exploratory and confirmatory factor analyses revealed a good factor structure that separated into two key themes: 1. Hoping and waiting for rescue from others and 2. Self‐reliance. Hoping and waiting for rescue was negatively correlated with self‐reliance. It was also correlated with parental over‐protection (but not care), lack of feeling socially safe, higher reassurance‐seeking, depression, anxiety, stress, and emotion dysregulation. Network analysis revealed a stable network in which hoping and waiting for rescue is a central node with direct connections to variables of mental health, social relating, and early life experiences. The scales demonstrated good test–retest reliability and internal consistency.

**Conclusions:**

This study suggests that individuals who feel they need others to rescue them from distressing mental states are less oriented to self‐reliance and self‐help. Moreover, this coping style is associated with a range of mental health difficulties. Therapists can be alert to these difficulties regarding why clients might not engage in self‐help and help clients address them, including linking them to other issues such as unprocessed emotions associated with early attachment difficulties.

## INTRODUCTION/BACKGROUND

There is a long history to the idea that to have a well‐functioning mind, we need to gain insight into its processes and train it with appropriate personal practices. This is the essence of the contemplative traditions (Hanh, 2008; Kelly, 2013). Indeed, all skills training requires practice. Guided self‐practice became central to the recently developed cognitive‐behavioural therapies in the forms of (for example), self‐awareness, mindfulness, assertiveness and thought reappraisal (Beck et al., 1979, 2015; Dobson & Dozois, 2021; Farrand & Woodford, 2013). In the compassion focused therapy approaches, various forms of mind training and personal practice are regarded as important constituents for change (Germer & Neff, 2021; Gilbert & Simos, 2022; Goleman & Davidson, 2017). Indeed, the last 30 years has seen a considerable development in the self‐help movement that seeks to provide people with information and practices that guide them through ways of helping themselves in areas such as diet, healthy living, relationships, emotion regulation, anxiety and depression, and ‘insight into the nature of one's own mind’. Unfortunately, some of these guides are not based on well‐informed research studies and, as Harwood and L'Abate (2009) indicate, can be more to do with selling products. The use of guided self‐help has also been partly pioneered because of the high numbers of people requiring help compared to the clinical resources available (Lewis et al., 2012; Lovell et al., 2006). This has resulted in the development of what is sometimes called low‐intensity cognitive behaviour therapy (CBT), which utilises many forms of guided self‐awareness and self‐help training (e.g. monitoring thought‐emotion links, mindfulness, reappraisal, metacognition, behavioural practice; Ridgway & Williams, 2011). However, even when people know what to do to help themselves, such as for weight, fitness, assertiveness, problem‐solving, and changing behaviours, that knowledge may not translate into behaviour change (Sheeran & Webb, 2016). Some people hope things will change by themselves. In addition, if practices do not work quickly or when people *fail* to make self‐help work, they can give up and come to believe they are unable to help themselves (i.e. Beatty & Binnion, 2016; Wojtowicz et al., 2013).

Macleod et al. (2009) found that self‐help materials were commonly used by CBT therapists (although only 38.2% were trained to do so). The helpfulness of these materials was linked to five factors: client motivation, the credibility of the materials, likely adherence, a sense of self‐efficacy, and a low degree of hopelessness. Farrand and Woodford (2013) also found that the type of support clients received with self‐help (e.g. face to face versus telephone contact) impacted its effectiveness. Dispositions to help oneself or rely on others can arise from early attachment experiences. For example, Bowlby (1980) described how people with anxious attachment could become overly dependent on others. In contrast, avoidant attachment styles can generate compulsive self‐reliance. Although neglect, high expressed emotion, and abuse ‐ impact attachment styles (Music, 2017; Cassidy & Shaver, 2016; Mikulincer & Shaver, 2023), another dimension that impacts people's beliefs and attitudes about their needs for others to rescue them is the degree of overprotection and early care (Parker et al., 1979). Berry et al. (2007) found significant associations between parental overprotection (but not parental care) and attachment anxiety (fear of being abandoned or losing others). This suggests that overprotection may undermine confidence and self‐reliance. Parental warmth has been found to play a major role in vulnerability to depression and anxiety (Richter et al., 2009; Santos et al., 2023) and may also play a role in facilitating self‐confidence or the need for others.

Most therapists have encountered individuals who seem to be waiting for somebody to come and rescue them, tell them what to do, or have ‘the answer’. Some hope that they will ‘one day’ meet someone who will rescue them from loneliness or a bad marriage, or find someone who understands, empathises, and cares about them. Some feel they are waiting to find their ‘soul mate’. Holmes (2014) highlighted that rather than looking for ways to help oneself, some clients are looking for forms of connectedness that give them a sense of a secure base and safe haven. Such hopes and searches are crucial to understanding some forms of therapy (Gilbert & Simos, 2022; Holmes & Slade, 2017). Others hope that they might find someone who will find out what is wrong with them and put them right; diagnose and ‘treat’ their condition (e.g. depression with drugs). These difficulties, resting on the fantasised hope of finding something outside of oneself, may interfere with efforts to engage with self‐help materials.

In contrast, some people prefer to be self‐reliant. Some may not trust others to either want to help or be able to help them. Some can avoid help‐seeking due to feelings of shame (Gulliver et al., 2010) and perceptions of stigma (Schibalski et al., 2017) including revealing what help is needed (Gilbert, 2017). Studies have shown this is especially the case for young people (Ishikawa et al., 2023; Spear & Kulbok, 2004). Jennings et al. (2015) found that self‐stigma was related to higher self‐reliance in college students and that this was related to a negative attitude toward treatment‐seeking for mental health difficulties and a decreased probability that those with mental health difficulties actually pursued treatment. Alongside the self‐help movement, there has been a major proliferation of successful medical interventions in the form of (for example) surgery and pharmacology. These not only treat various disorders (e.g. infections, diseases and injuries) but can also reduce pain and disability. Indeed, we live in a world of extraordinary advances and ‘miracles’ in biomedicine, and certainly one of the authors would not be here today without it. In addition, the emergence of psychotropic drugs marketed to reduce anxiety and depression adds to the belief that solutions to one's mental states depend on getting the ‘right treatment’. This clearly sets a cultural zeitgeist of dependency on others, since we cannot do surgery on ourselves or make our own analgesics, antibiotics, or vaccines.

### Evolution and waiting for help

These difficulties of struggling to help people with self‐practice have been noted in many therapies. As discussed above, waiting for help to arrive rather than trying to actively help oneself is different. In addition, the phenomenology of waiting for rescue can also point to the activation of evolved defence strategies. For example, individuals who have been confronted with uncontrollable stress can become passive and helpless and give up trying to help themselves, even when they can (Seligman, 1975). Some may feel defeated after repeated struggles (Gilbert & Allan, 1998; Siddaway et al., 2015). This is particularly important given that defeat states are linked to anhedonia and reduced drive (Gilbert et al., 2002).

Gilbert (1992, 2022) noted that hoping and waiting for rescue may link to the evolution of attachment processes and defences. The evolution of the attachment relationship between parent and offspring enabled the parent to be both a provider of resources and a protector of infants. Mammalian offspring begin life being totally dependent on others, orientated to seek out and need the inputs of others. Hence, infants evolved to stay close to parents (Bowlby, 1969, 1973, 1980; Cassidy & Shaver, 2016; Holmes & Slade, 2017; Mikulincer & Shaver, 2023). In the advent of separation, and unavailability of substitute carers, mammalian infants first show invigorated activity to reunite, called ‘protest’, but if this activity and movement fails then the infant is at risk of getting lost and attracting predators. Hence, infants need to reduce their movement in the environment and ‘wait to be found and rescued’ (Bowlby, 1969, 1973, 1980; Cassidy & Shaver, 2016). What is crucial to the attachment context is that ‘in the wild’ it is actually dangerous to continue to be proactive, go searching and move. Indeed, their own activity needs to be closed down (Bowlby, 1980; Cassidy & Shaver, 2016; Gilbert, 1988, 1989, 2000). This defence strategy shifts offspring from activation of searching and calling to deactivation of self‐initiated behaviour, i.e. waiting for a parent/carer to find and help them. One research question is the degree to which this attachment defence response pattern has been sensitised in childhood and is easily triggered later in life. Like learned helplessness, when confronted by stress there may be an automatic triggering of the psychophysiological mechanisms of these defences giving rise to ‘a felt sense’ that one needs others to rescue them.

Hoping and waiting for rescue is a different concept to that of seeking social support. Taylor (2006) says: “Social support is defined as the perception or experience that one is loved and cared for by others, esteemed and valued, and part of a social network of mutual assistance and obligations” (p. 192). In contrast, hoping and waiting for rescue depicts a passive sense of waiting for solutions to address suffering and difficulties, waiting for ‘something to happen’, ‘things to just change’ or for a ‘rescuer/saviour’ to arrive. It is also a different concept to reassurance seeking (RS). Radomsky et al. (2020) says “conceptualizations of RS across disorders suggest that it is a repetitive safety‐seeking behaviour following perceived general or social/relational threats, despite having received the information before (e.g. ‘Are you really sure the door is locked?’, ‘Are you sure you still love me?’” (p. 2). Hoping and waiting for rescue is different because individuals are ‘waiting’, they are not actively seeking, requesting or doing things to elicit help. People can get stuck in ‘reassurance traps’ which can lead to “paralysis in decision‐making, haunting worries about making a mistake or causing harm, insecurity and self‐doubt” (Winston & Seif, 2022). However, there can be overlap between tendencies to seek reassurance and waiting for rescue in that both may be associated with behavioural inaction. Additionally, both concepts rely on help from others rather than that which is self‐directed.

### Aims

Given the above processes that could underpin a closing down of self‐help orientation, this study sought to develop a self‐report scale that asked specific questions about hoping and waiting for rescue. Assuming the scale has adequate psychometric properties, we also wanted to explore these processes in relation to early life history, depression, anxiety, and stress, emotion regulation, self‐other relating, and reassurance‐seeking.

## METHODS

### Ethics

This was sought from the University of Derby ethics committee and granted on 20th February 2023. The committee did suggest that we focus on ‘hoping’ for rescue when presenting the scale to participants, rather than ‘waiting’. The problem with that, however, is that it does not capture the waiting and passivity aspect. So, although we followed the ethics committee’s advice on this occasion and did not suggest changing item wording, we personally believe that the scale should be recognised as a measure of hoping *and waiting*. Hence, we refer to it as hoping and waiting in this write‐up.

### Participants

This study recruited two main samples of participants from the UK and two smaller samples to assess test–retest reliability. Participants from all samples were recruited and paid via the online survey platform, Prolific (www.prolific.co), from which they were redirected to Qualtrics (Qualtrics, Provo, UT) where the study materials were hosted. All data were collected between April 2023 and March 2024.

The first sample (*n* = 250) were recruited to investigate the process of hoping and waiting for rescue, how it relates to study variables, and to explore the factor structure via exploratory factor analysis (EFA). In this sample, 5 participants were identified statistically as outliers in more than one variable and were removed from the dataset. The final sample (*n* = 245) consisted of 67 males (27.3%), 176 females (71.8%), and 2 who selected ‘prefer not to say’ (0.8%). Ages ranged from 18 to 78 years (*M* = 39.66, *SD* = 13.72).

The second sample (*n* = 200) were recruited to further explore how hoping and waiting for rescue related to other study variables and to conduct a confirmatory factor analysis (CFA). This sample comprised 100 males (50%), 98 females (49%) and two who selected ‘prefer not to say’ (1%), with ages ranging from 18 to 78 (*M* = 39.64, *SD* = 12.28). There were no statistical outliers in this sample.

Two test–retest samples were recruited to assess the test–retest reliability of the scale. One sample (*n* = 40) completed the scale at two timepoints separated by 2–3 weeks. This sample comprised 15 males (37.5%) and 25 females (62.5%) and ages ranged between 20–69 (*M* = 39.63, *SD* = 14.92). Another test–retest sample (n = 40) was recruited with a longer interval between administrations of 6–7 weeks. Thirty‐three participants completed both parts of the study; of these, 11 were male (33.3%), 22 were female (66.6%) and ages ranged from 18 to 67 (*M* = 41.06, *SD* = 14.51).

### Procedure

All participants were provided with information about the study via Prolific. If they decided to participate, they were then provided with an information sheet and consent form via Qualtrics and asked to create a unique ID code so that they had the option to withdraw their data within 4 weeks of participation. After providing informed consent, they were asked to complete a demographics form which asked for their age and sex.

The main study participants (total *n* = 445) completed the new hoping and waiting for rescue scale along with other established self‐report scales measuring: (1) emotion regulation, (2) reassurance seeking, (3) perception of the self in relation to others, (4) social safeness, (5) social comparison, (6) early memories of warmth and safeness, (7) parental bonding, and (8) depression, anxiety, and stress. This was estimated to take 25–30 minutes, and participants were paid £6.00 for their time upon completion.

Both test–retest samples (total *n* = 73) completed the hoping and waiting for rescue scale and were then redirected to a separate Qualtrics form and asked for their email address, so that they could be sent an automated email invitation to participate in the second part of the study either 2 or 6 weeks later. After this interval, they were asked to complete the hoping and waiting for rescue scale again. Completing the scale at both time points was estimated to take less than 10 minutes, and participants were paid £3.00 upon completion. All participants were debriefed after the study.

### Measures

#### Hoping and waiting for rescue and self‐reliance scales

The two new measures developed in this study were initially administered as part of a single, 18‐item scale with a Likert scoring format to measure the extent to which people hope and wait for others to rescue them. Eight of the items were initially designed as reverse filler items. However, the use of reverse items with the assumption that the dependent variable is made up of a single dimension from positive to negative is potentially problematic. Therefore, they were regarded as potentially separate scales and were explored using factor‐analytic methods.

Participants were prompted with the statement ‘when I'm feeling down, stressed or distressed…’ and asked to rate each item from 1–5 (1 = not at all like me, 2 = a little bit like me, 3 = moderately like me, 4 = quite a bit like me and 5 = extremely like me). Hoping and waiting for rescue items include: “I yearn for someone to come and help me” and “I feel I am waiting for someone to take control and sort things out”. The self‐reliance items include: “I prefer to help myself rather than wait for others to help me” and “I like to keep my problems private and sort them out myself”. The full scale can be found at https://www.compassionatemind.co.uk/resource/scales.

#### Brief version of the difficulties in emotion regulation scale (DERS‐16; Bjureberg et al., 2016)

The DERS is a 36‐item scale measuring difficulties in emotion regulation across six categories: nonacceptance of negative emotions; inability to engage in goal‐directed behaviours when distressed; difficulties controlling impulsive behaviours; limited access to emotion regulation strategies perceived as effective; lack of emotional awareness, and lack of emotional clarity (Shahabi et al., 2020). A short version of the scale comprising 16 items was developed by Bjureberg et al. (2016). As in the original measure, participants indicate on a 5‐point Likert scale (1 = almost never; 5 = almost always) the extent to which each statement applies to them. Items include: “I have difficulty making sense out of my feelings” (clarity) and “when I am upset, I start to feel very bad about myself” (strategies). The 16‐item version shows good internal consistency (*α* = .92), good test–retest reliability, and good convergent and discriminant validity. In addition, the DERS‐16 shows minimal differences in convergent and discriminant validity when compared to the original DERS.

#### The covert and overt reassurance‐seeking inventory (CORSI; Radomsky et al., 2020)

The CORSI is a 26‐item scale which measures five types of reassurance‐seeking: overt social relational threat; covert social relational threat; overt general threat; covert general threat (active) and covert general threat (passive) (Radomsky et al., 2020). Items include “I sometimes threaten to end a friendship in order to see if my friends really care for me” and “when faced with an important decision, I need to ask others for reassurance before I can make my final choice”. Participants rate each item on a 5‐point Likert scale (from 0 ‘not at all’ to 4 ‘very much’). The measure shows excellent internal consistency in a clinical sample (*α* = .93) and in a nonclinical (student) sample (*α* = .93) (Radomsky et al., 2020). At present, there is no test–retest reliability data available for this scale.

#### The self‐other scale (SOS; Dagnan et al., 2002)

The SOS is a 14‐item scale designed to measure people's sense of themselves in relation to others (Dagnan et al., 2002). While some people feel they need to be close to others to feel a sense of security, others can feel threatened or controlled and want distance between themselves and others. Scale items belong to one of two factors: insecurity, e.g. “when I am alone I feel the need to contact somebody” or engulfment, e.g. “I can feel suffocated if I am too close to someone”. Responses are recorded on a Likert scale from 1 ‘almost never’ to 5 ‘very often’. The insecurity and engulfment subscales show good internal reliability (*α* = .76 and .82 respectively) and good test–retest reliability (Dexter‐Smith et al., 2003). Dagnan et al. (2002) reports good internal consistency for the scale as a whole (*α* = .84).

#### Social safeness and pleasure scale (SSPS; Gilbert et al., 2009)

The SSPS (Gilbert et al., 2009) assesses the extent to which individuals feel a sense of warmth, acceptance and connectedness in their social world. The scale consists of 11 statements which are grouped into a single factor. Participants rate each statement using a Likert scale ranging from 1 (‘almost never’) to 5 (‘almost all the time’). Items include: “I feel a sense of belonging”, “I feel secure and wanted” and “I feel accepted by people”. The scale initially showed excellent internal reliability (*α* = .92; Gilbert et al., 2009) and Miguel et al. (2022) recently corroborated this in a study with adolescent participants (*α* = .93; Miguel et al., 2022). Kelly et al. (2012) found that social safeness was more strongly related to numerous indicators of vulnerability and psychopathology than affect (positive/negative) and perceived social support. It has been used in a range of studies and there is indication that social safeness may be an emotion system in its own right.

#### Social comparison scale (Allan & Gilbert, 1995)

The SCS is an 11‐item scale measuring global comparisons of self with others. Items are organised into three categories: attractiveness, rank and group fit (Allan & Gilbert, 1995). It utilises a semantic differential methodology in which participants respond on a 10‐point bipolar scale to questions of comparison, e.g. ‘incompetent’/‘competent’; ‘untalented’/‘more talented’; ‘undesirable’/‘more desirable’, following the prompt “in relationship to others I feel…”. Low scores indicate feelings of inferiority and low‐rank self‐perception. Allan and Gilbert (1995) reported that the scale has good internal consistency within clinical populations (*α* = .88 and .96) and student populations (*α* = .91 and .90) and significant correlations with measures of psychopathology, including depression.

#### Early memories of warmth and safeness scale—brief version (EMWSS‐B; Vagos et al., 2017)

The EMWSS‐B is a short, 9‐item version of the original 21‐item EMWSS (Richter et al., 2009) which is a self‐report scale measuring recall of feeling safe and cared for, and of receiving emotional warmth in early childhood. Items include: “I felt that I was a cherished member of my family” and “I had feelings of connectedness” which participants rate on a 5‐point Likert scale (0 = ‘No, never’; 1 = ‘Yes, but rarely’; 2 = ‘Yes, sometimes’; 3 = ‘Yes, often’; 4 = ‘Yes, most of the time’). Richter et al. (2009) reported that the scale shows excellent internal consistency (*α* = .97) and Vagos et al. (2017) reported a similar Cronbach's alpha for the brief version of the scale (*α* = .96).

#### Parent bonding instrument (PBI; Parker et al., 1979)

The PBI is a 25‐item retrospective self‐report scale which measures parenting styles as perceived by the child. Respondents complete the scale separately for their mother and father. The measure consists of two subscales: ‘care’ (12 items) and ‘overprotection’ (13 items). Participants respond to statements such as [mother/father] “did not help me as much as I needed” and [mother/father] “tried to control everything I did” on a 4‐point Likert scale ranging from ‘very like’ to ‘very unlike’. Total scores for the care and overprotection subscales may be calculated, or alternatively parents may be assigned to one of four categories: affectionate constraint, affectionless control, optimal parenting and neglectful parenting. Several studies have found the PBI to have good test–retest reliability, even over an extended (20‐year) time period (Wilhelm et al., 2004).

#### Depression, anxiety, and stress scale (DASS‐21; Lovibond & Lovibond, 1995)

The DASS‐21 is a short 21‐item version of the DASS‐42 which measures depression, anxiety and stress. Items include: “I felt that I had nothing to look forward to”, “I tended to overreact to situations” and “I felt scared without good reason”. Participants are asked to rate each item on a 4‐point Likert scale ranging from 0 (‘Did not apply to me at all’) to 3 (‘Applied to me very much, or most of the time’) according to how much each statement applied to them over the past week. The DASS‐21 shows good to excellent internal consistency, with Cronbach's alphas of .88 (depression), .82 (anxiety), .90 (stress) and .93 for the scale as a whole (Henry & Crawford, 2005).

### Data analysis

Analyses were conducted using IBM SPSS version 28, IBM SPSS AMOS version 26, and JASP version 0.18.3.0. *Z*‐scores, scatterplots, and boxplots were inspected for the presence of outliers. Normality was assessed using skewness and kurtosis values, which were all within the recommended parameters of 2 for skewness and 7 for kurtosis (West et al., 1996). In the first sample, skewness values ranged from −0.78 to 1.6, and kurtosis values ranged from −1.16 to 1.73. In the second sample, skewness ranged from −0.77 to 1.66, and kurtosis ranged from −0.04 to −1.01. Histograms were also visually inspected as another indication of distribution. Descriptive statistics (means and standard deviations) and Cronbach's alpha values were calculated to explore the data for each variable and establish the reliability of the scales.

An exploratory factor analysis (EFA) was conducted using Maximum Likelihood extraction with Direct Oblimin rotation to establish the factor structure of the scales. The Kaiser‐Meyer‐Olkin (KMO) value was above the 0.8 parameter recommended for factor analysis. Results of the EFA were interpreted by examining the scree plot, total variance explained (%), communalities, factor loadings, and item correlations. A CFA was used to confirm the factor structure, and model fit was evaluated using the following indices and cut‐points indicating good fit (Kline, 2005; Tabachnick & Fidell, 2013). Normed Chi‐Square (*χ* ^2^/df) values between 2 and 5; Root Mean Square Error of Approximation (RMSEA) between 0.05 and 0.08; Standardized Root Mean Square Residual (SRMR) < 0.08; Tucker‐Lewis Index (TLI) and Comparative Fit Index (CFI) > 0.9.

Following confirmation of the factor structure, *t*‐tests were performed for each study variable to check for differences between the samples. All *t*‐tests were non‐significant, indicating that the data was not significantly different between the two main samples and that it would be appropriate to combine them in subsequent analyses. Pearson product–moment correlations were generated using the combined sample to explore the relationships between the two scales and other study variables and to establish convergent and discriminant validity. Intraclass correlation coefficients were used to indicate test–retest reliability, which showed the relationship between paired data at two time points with a 2–3 or 6–7 week interval.

Network analyses were conducted to provide a graphical illustration of how variables (or ‘nodes’) within the network relate to each other, the direction and strengths of these associations, and how they are situated within the wider network of factors. JASP network analyses and output are based on the R packages ‘bootnet’ (Epskamp et al., 2017) and ‘qgraph’ (Epskamp et al., 2012). The analysis was split by sample to explore similarities and differences. The EBICglasso estimator was used to provide estimates of the strength of associations between variables whilst controlling for the effects of other measured factors in the network. A tuning parameter (*λ*; set between 0–1) is applied to create a sparser network and minimise the number of spurious connections (Hevey, 2018; Epskamp et al., 2017). In this study, *λ* was set at a more liberal value of 0.25 to maximise the number of true edges; however, after comparing results when using a more stringent parameter of 0.50, there were no differences in the number of estimated edges. The network layout was set using the pre‐defined Fruchterman‐Reingold algorithm, which organises the network according to the strength of the ‘edges’ (connections) between nodes. Centrality measures of betweenness, closeness, and strength were calculated. Both edge and centrality stability were assessed using 1000 bootstrapped samples; nonparametric bootstrapping was used to indicate edge stability, whilst a case‐drop bootstrap was used to estimate centrality stability (Epskamp et al., 2017; Burger et al., 2023). All stability estimates were explored using the combined sample (*n* = 445) given the similarity between networks.

## RESULTS

### Exploratory factor analysis

An EFA was conducted on the whole scale using maximum likelihood estimation with direct oblimin rotation. The KMO measure of sampling adequacy statistic was 0.91, and Bartlett's test of sphericity was significant (*p* < .001) suggesting the data was suitable for EFA. Hair et al. (1998) recommended that the cut‐off point for factor loadings with a sample size of 250 should be 0.35; therefore, this criterion was applied in the interpretation of factors.

The initial EFA produced 3 factors with eigenvalues greater than 1, which explained 67.14% of the variance. Item 4 produced a low communalities value (<0.4), low factor loading (<0.35), and cross‐loaded on two factors; therefore, it was removed from subsequent analyses. The second EFA produced 2 factors explaining 63.35% of the variance; however, item 13 had a low communalities value and weak factor loading and was removed. The final EFA included 10 hoping and waiting for rescue items and 6 self‐reliance items belonging to two factors, which explained 64.72% of the variance. Factor loadings ranged between 0.54 and 0.89 and are shown in Table 1.

**TABLE 1:** Items, factor loadings, and variance explained (%) for the hoping and waiting for rescue factor.

Item number	Item	Factor loading	Corrected item‐total correlations
9	I feel I'm waiting for someone to come and care for me	0.89	0.87
12	I feel I need to be rescued in some way	0.85	0.83
6	I feel I need someone to come and ‘find me’	0.84	0.82
8	I yearn for someone to come and help me	0.83	0.80
10	I feel someone out there could help me, I just have to wait for them	0.78	0.73
14	I feel I am waiting for someone to take control and sort things out	0.77	0.77
16	I daydream about someone coming to help me	0.76	0.72
5	I feel detached as if I am waiting for something or someone	0.72	0.66
3	I feel unable to help myself	0.65	0.67
2	I am waiting for others to help me feel better	0.55	0.62
Variance explained (%)		45.57%	

Items and factor loadings for each scale are shown in Tables 1 and 2.

**TABLE 2:** Items, factor loadings, and variance explained (%) for the self‐reliance factor.

Item number	Item	Factor loading	Corrected item‐total correlations
18	I tend to wait until I just get over it on my own	0.82	0.70
17	I like to keep my problems private and sort them out myself	0.79	0.67
7	I am better relying on myself rather than others	0.77	0.73
1	I prefer to help myself rather than wait for others to help me	0.72	0.71
11	I prefer working on myself rather than seeking help	0.72	0.72
15	I work hard to try to help myself	0.54	0.55
Variance explained (%)		19.15%	

### Confirmatory factor analysis

A CFA of the proposed factor structure using data from the second sample (*n* = 200) was conducted using SPSS AMOS. Model fit indices initially suggested a poor fit to the data *χ* ^2^/df = 3.418, *p* < .001; TLI = 0.849; CFI = 0.87; RMSEA = 0.11, *p* < .001, SRMR = 0.095.

The model was respecified by correlating the error terms of several items. Modification indices (values >11) suggested that items 3 and 15, 17 and 18, 3 and 9, and 5 and 9 should be correlated. Error terms were only correlated if doing so was both statistically and theoretically justified in order to avoid purely statistically driven model fitting.

**FIGURE 1:**
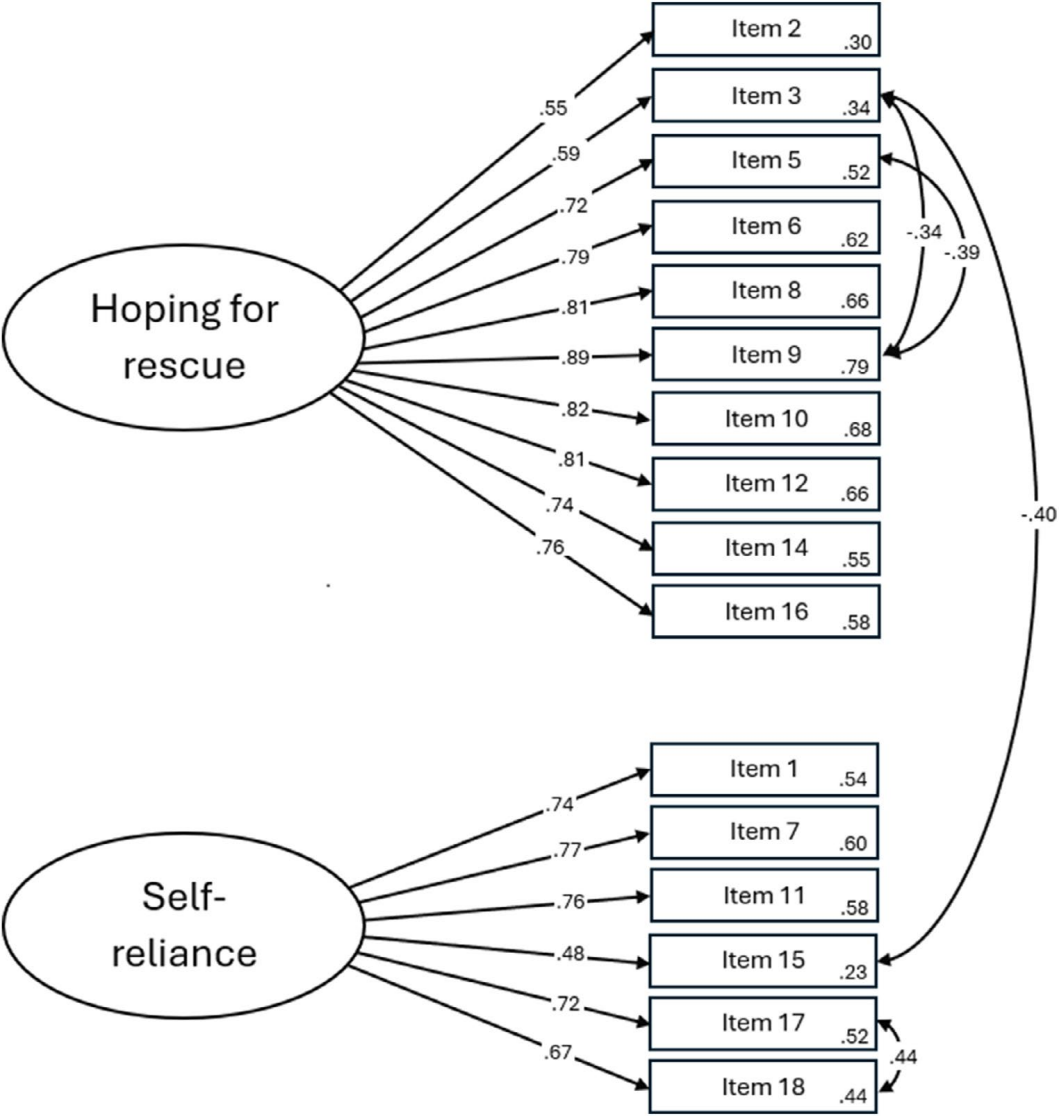
Respecified CFA model with four pairs of error terms correlated.

The respecified model, with four pairs of error terms correlated, showed adequate fit to the data, *χ* ^2^/df = 2.514, *p* < .001; TLI = 0.906; CFI = 0.922; RMSEA = 0.087, *p* < .001, SRMR = 0.085 (Figure 1).

Descriptives and internal consistency statistics (Cronbach's alphas) for all variables are shown in Table 3.

**TABLE 3:** Means, standard deviations, and Cronbach's alphas for each study variable in the combined sample (*n* = 445).

	*M*	*SD*	Cronbach's alpha (*α*)
Hoping and waiting for rescue	19.96	9.11	.93
Self‐reliance	22.63	5.26	.87
Maternal care	25.06	8.93	.95
Maternal overprotection	14.24	7.68	.89
Paternal care	20.01	9.95	.95
Paternal overprotection	11.91	7.45	.88
Early memories of warmth and safeness	22.76	9.70	.96
Social safeness and pleasure	38.46	11.06	.97
Depression	6.37	6.04	.94
Anxiety	4.14	4.13	.85
Stress	7.00	5.26	.91
Difficulties in emotion regulation (DERS)	39.14	16.69	.96
Nonacceptance of negative emotions	7.33	3.51	.88
Inability to engage in goal‐directed behaviours	9.01	3.71	.92
Impulse control difficulties	6.12	3.44	.91
Limited emotion regulation strategies	12.39	5.95	.93
Lack of emotional clarity	4.29	2.24	.94
Social comparison	59.29	18.21	.93
Reassurance‐seeking (CORSI)	30.01	19.13	.95
Overt social relational	1.47	2.23	.77
Covert social relational	8.91	6.42	.89
Overt general	10.17	7.58	.92
Covert general—active	5.71	2.91	.52 (if item 1 removed, .69)
Covert general—passive	3.75	3.22	.77
Insecurity threat to self‐construction (SOS)	16.92	5.69	.83
Engulfment threat to self‐construction (SOS)	19.26	6.61	.86

Mean scores for males and females for each scale are shown in Table 4. Levene's test for equality of variances was non‐significant for both scales; therefore, independent samples *t*‐tests with equal variances assumed were performed. Results showed no significant differences between males and females for either scale.

**TABLE 4:** Means and standard deviations for each sex, for each subscale, including *t*‐test results.

Scale	Male		Female		*t*	*p*
	*M*	*SD*	*M*	*SD*		
Hoping and waiting for rescue	19.45	9.04	20.15	9.09	−0.79	.43
Self‐reliance	22.80	5.33	22.56	5.24	0.46	.65

### Test–retest reliability

The test–retest reliability of the scales was examined in two separate UK samples recruited via Prolific. The first sample (*n* = 40), recruited in July 2023, completed the scale at two time points with an interval of 2–3 weeks, whilst the second sample (*n* = 33), recruited in January 2024, completed the scale with an interval of 6–7 weeks.

The intraclass correlation coefficient (ICC) for each sample was calculated using a two‐way mixed model with absolute agreement to estimate the stability of the participants' scores. The ICC between scale scores with a 2–3 week interval was 0.88 for the hoping and waiting for rescue scale and 0.75 for the self‐reliance scale, indicating good to excellent reliability. Analysis of the second test–retest data with a 6–7 week interval produced an ICC of 0.88 for hoping and waiting for rescue and 0.83 for self‐reliance, indicating good to excellent reliability.

### Correlations, convergent and divergent validity

To explore how hoping and waiting for rescue and self‐reliance relate to other study variables, and to establish convergent and discriminant validity, Pearson product–moment correlations were generated from the combined sample. All correlations are shown in Table 5.

**TABLE 5:** Pearson product–moment correlations for hoping‐waiting for rescue and self‐reliance with other study variables.

	Pearson's *r*	
	Hoping and waiting for rescue	Self‐reliance
Hoping and waiting for rescue	–	−0.33**
Self‐reliance	−0.33**	–
Maternal care	−0.16**	−0.11*
Maternal overprotection	0.23**	0.04
Paternal care	−0.08	−0.06
Paternal overprotection	0.20**	0.03
Early memories of warmth and safeness	−0.25**	−0.05
Social safeness and pleasure	−0.44**	−0.00
Social comparison	−0.40**	0.10*
Reassurance‐seeking (CORSI)	0.59**	−0.18**
Overt social‐relational	0.49**	−0.17**
Covert social‐relational	0.61**	−0.20**
Overt (general)	0.60**	−0.26**
Covert (general—active)	0.22**	0.08
Covert (general—passive)	0.37**	−0.03
Insecurity threat to self‐construction (SOS)	0.51**	−0.32**
Engulfment threat to self‐construction (SOS)	0.35**	0.19**
Depression	0.56**	−0.11*
Anxiety	0.47**	−0.13**
Stress	0.50**	−0.09
Difficulties in emotion regulation (DERS)	0.64**	−0.20**
Nonacceptance of negative emotions	0.51**	−0.11*
Inability to engage in goal‐directed behaviours	0.55**	−0.20**
Impulse control difficulties	0.58**	−0.20**
Limited emotion regulation strategies	0.63**	−0.20**
Lack of emotional clarity	0.51**	−0.17**

* Significant at .05 level.

** Significant at .01 level.

Hoping and waiting for rescue was significantly correlated with all measures, with the exception of paternal care. Analyses revealed strong, positive correlations with reassurance‐seeking, insecurity threat, depression, stress, and difficulties in emotion regulation. Hoping and waiting for rescue was also moderately correlated with anxiety and (negatively) with social safeness and social comparison.

Self‐reliance was significantly negatively correlated with insecurity threat, emotion dysregulation, reassurance‐seeking, anxiety, depression, and maternal care. It was positively correlated with engulfment threat and social comparison.

Reassurance‐seeking shows a strong, positive association with hoping and waiting for rescue, indicating convergent validity. It also negatively (although weakly) correlates with self‐reliance, indicating some degree of divergent validity with that construct. Further evidence of convergent/divergent validity is indicated by positive and negative associations with emotion dysregulation.

### Multiple regression

Multiple regressions were conducted to explore which independent variables predicted: (1) hoping and waiting for rescue, (2) self‐reliance, and (3) difficulties in emotion regulation. All assumptions for multiple regression analysis were met.

In the hoping and waiting for rescue model, the following predictors were used: reassurance‐seeking, difficulties in emotion regulation, depression, and insecurity threat. For the self‐reliance model, predictors were: difficulties in emotion regulation, reassurance‐seeking, insecurity threat, and hoping and waiting for rescue. In the emotion dysregulation model, the predictors used were: social comparison, reassurance‐seeking, depression, anxiety, stress, and hoping and waiting for rescue.

Regarding hoping and waiting for rescue, the model accounted for 52% of the variance (*F* = 119.22, *p* < .001). Depression emerged as the most powerful predictor (*β* = .25; *p* < .001), followed by emotion dysregulation (*β* = .23; *p* < .001), insecurity threat (*β* = .22; *p* < .001) and reassurance‐seeking (*β* = .20; *p* < .001).

For self‐reliance, the model accounted for 14% of the variance (*F* = 19.43, *p* < .001). Hoping and waiting for rescue was the most powerful predictor (*β* = −.29; *p* < .001), followed by insecurity threat (*β* = −.25; *p* < .001). Emotion dysregulation and reassurance‐seeking did not emerge as significant predictors.

In a third analysis with emotion dysregulation as the dependent variable, the model accounted for 69% of the variance (*F* = 163.87, *p* < .001). The strongest predictor was stress (*β* = .27; *p* < .001), followed by reassurance‐seeking (*β* = .21; *p* < .001), hoping and waiting for rescue (*β* = .20; *p* < .001), depression (*β* = .15; *p* < .001), social comparison (*β* = −.13; *p* < .01) and anxiety (*β* = .09; *p* < .05).

### Network analysis

**FIGURE 2:**
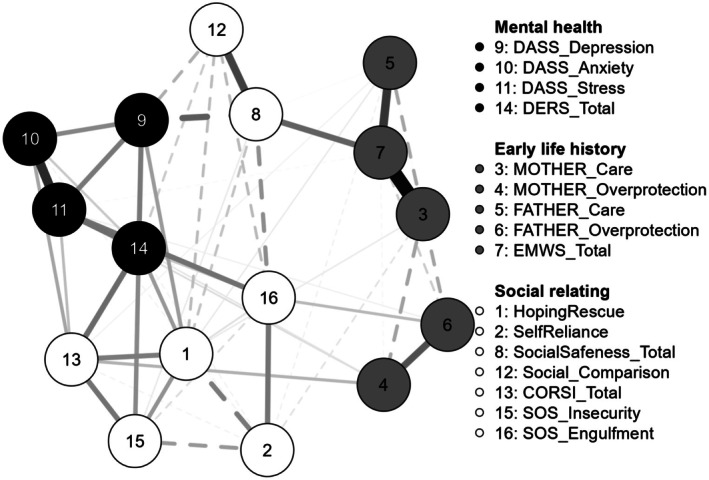
Network plot for sample 1.

**FIGURE 3:**
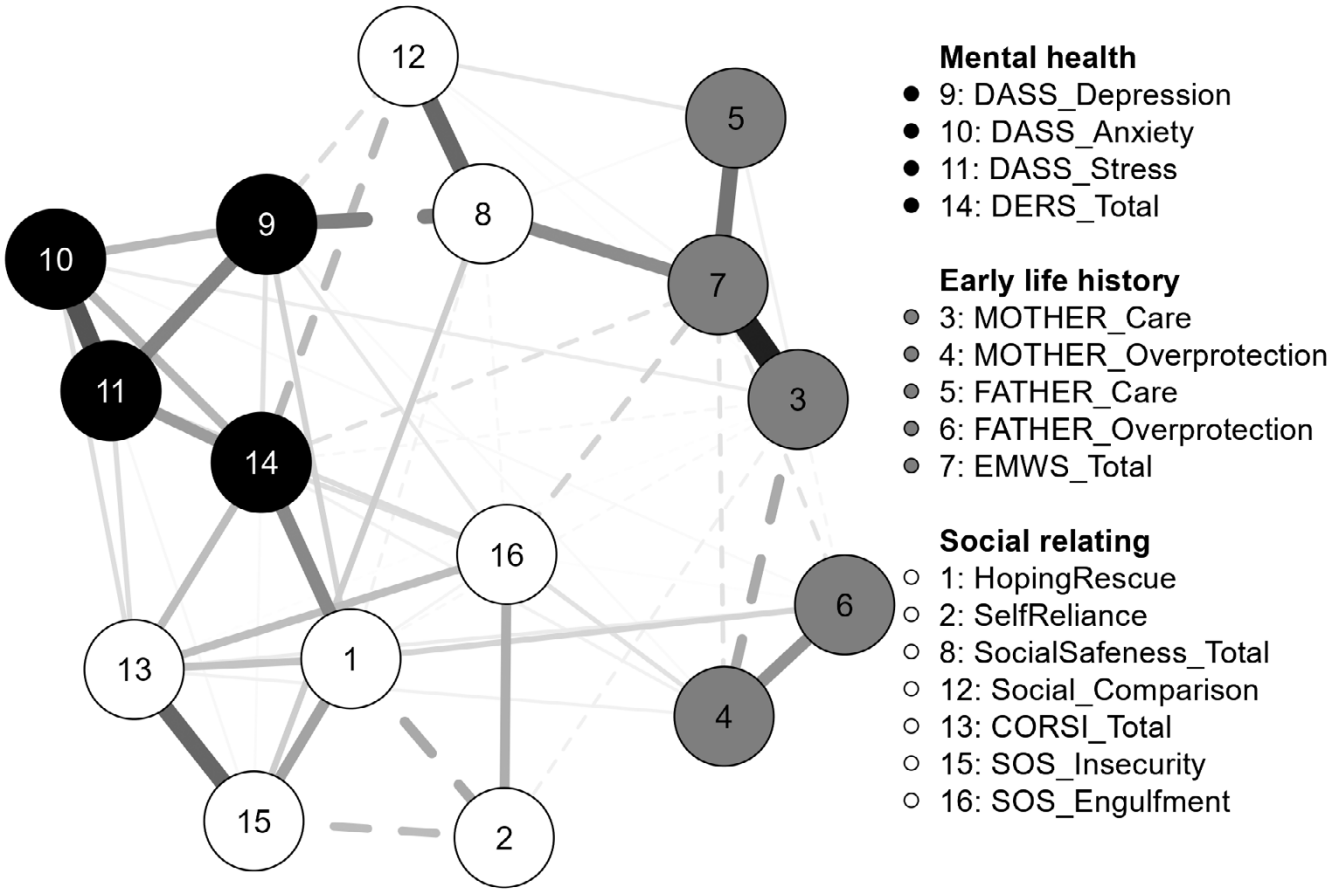
Network plot for sample 2.

A network analysis was conducted using all study variables, with participants split by sample. Network plots of study variables used data from sample one (*n* = 245) and sample two (*n* = 200). Solid lines indicate positive relationships, and dashed lines indicate negative relationships. Thicker and darker lines indicate stronger relationships. The network of sample one is in Figure 2, and sample two is in Figure 3.

The network of sample one had a density of 0.54 (55/120 non‐zero edges) with a mean edge weight of 0.029. Sample two produced a network with a density of 0.51 with 59/120 non‐zero edges and a mean edge weight of 0.032. The largest edge weight was found between early memories of warmth and safeness and maternal care (0.62 in sample one; 0.55 in sample two). The smallest edge weight was found between maternal and paternal care in sample one (0.004) and between maternal care and reassurance‐seeking in sample two (−0.004). Hoping and waiting for rescue is centered between variables of self‐reliance, insecurity threat, reassurance‐seeking, emotion dysregulation, and engulfment threat. Hoping and waiting for rescue had the strongest connection with self‐reliance in sample one (−0.20) and with emotion dysregulation in sample two (0.28). Early life history variables appear connected to the rest of the network largely via social safeness (node 8).

Edge stability was assessed using a nonparametric bootstrap procedure with 1000 samples. Overall, these samples appear closely related to the original, suggesting edge weights are stable. Although several of the 95% confidence intervals overlap with zero, in the case of regularized partial correlation networks, it should not be assumed that they cannot be differentiated from zero (Fried, 2018).

**FIGURE 4:**
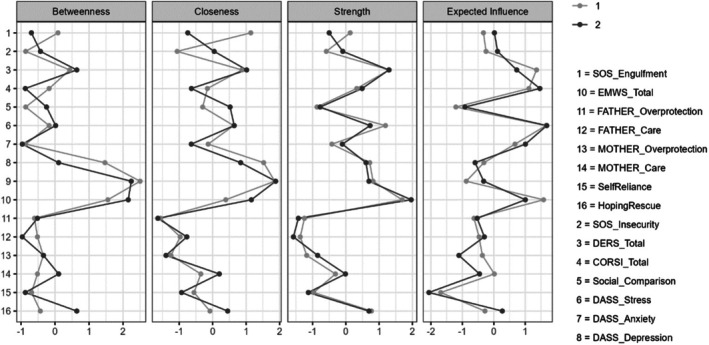
Centrality plots showing indices of betweenness, closeness, and strength for each study variable, split by sample (‘1’ and ‘2’).

Figure 4 shows the centrality plots for betweenness, closeness, and strength for each node. ‘Betweenness’ refers to how often a node lies on the shortest path between other nodes. ‘Closeness’ indicates how well a node is indirectly connected with other nodes. ‘Strength’ indicates how strongly a node is directly connected to other nodes. Strength centrality indicated early memories of warmth and safeness (node 10) to be the most strongly connected node within the network in both samples, while paternal care (node 12) and paternal overprotection (node 11) were indicated to be the weakest nodes.

Centrality stability was assessed using 1000 case‐drop bootstrap samples, which indicated that strength centrality showed the strongest correlation with the original sample, although all centrality indices were strongly correlated with original estimates (>0.75) even after dropping 70% of cases from the dataset. Centrality statistics, stability plots, and stability statistics can be found in Supporting Information.

#### Network comparison between samples

To compare networks between samples, average absolute edge weights for all variables were correlated between samples one and two. Edge weights for thirteen of the sixteen variables were very strongly correlated between samples (Pearson's *r* > 0.84). Correlations were weaker for reassurance‐seeking (*r* = 0.76), emotion dysregulation (*r* = 0.75) and engulfment threat (*r* = 0.57) suggesting these variables have slightly less stable associations with other variables in the network.

## DISCUSSION

### Validity of new scales

The factor analysis suggested that the items in the hoping and waiting for rescue and self‐reliance scales represented two separate factors. Each factor is valid and coherent with good Cronbach alphas. Both subscales showed good test–retest reliability; after 6–7 weeks, the intraclass correlation coefficients were 0.88 for hoping and waiting for rescue and 0.83 for self‐reliance. The 2–3 week test–retest study indicated that self‐reliance may be more time‐variant. Hoping and waiting for rescue and self‐reliance were negatively correlated at *r* = −0.33. The fact that this correlation is not higher could be because people with secure attachment may be appropriately self‐reliant but can also recognize when they need to wait for others to help them. In other words, these are context‐dependent processes, i.e. one may be self‐reliant in some areas (e.g. personal finances) but passive in others (e.g. waiting for an operation). Table 5 shows the Pearson correlations for all variables. In regard to early life history, maternal care had a small negative correlation with hoping and waiting for rescue, while maternal and paternal *over‐protection* were both significantly positively correlated with hoping and waiting for rescue. Similar findings were reported by Berry et al. (2007) where parental overprotection was significantly associated with anxiety in attachment relationships. There was also a significant negative correlation (−0.25) between hoping and waiting for rescue and experiences of early warmth and feeling safe.

### Social context

Hoping and waiting for rescue was negatively correlated with social safeness at −0.44. This is interesting because one could have made the opposite prediction; that feeling socially safe means one can trust others to be supportive and therefore wait for them to help. This finding, however, indicates that individuals who do not feel safe in their current context may feel a lack of availability of potential helpers but hope they will turn up or come into their social network.

### Social comparison

One reason that people may look to others for help is that they lack confidence in themselves or feel inferior. We explored this in relation to social comparison, and indeed there is a moderate correlation between people who are hoping and waiting for rescue and feeling inferior compared to others (*r* = −0.40). However, social comparison was not related to self‐reliance. In other words, self‐reliance seems to be independent of or concerned with social comparison.

### Reassurance‐seeking

A process that might appear to be close to hoping and waiting for rescue is reassurance‐seeking. Although, as noted in the introduction, reassurance‐seeking is a much more active process than the passivity of hoping and waiting for rescue, it did correlate at *r* = 0.59. All subfactors of reassurance‐seeking significantly correlated with hoping and waiting for rescue but either not at all or minimally with self‐reliance. Clearly then, the need for reassurance is part of the experience of waiting for others to come and help. Especially noteworthy is the covert social relational variable that correlates with hoping and waiting for rescue at *r* = 0.61 whereas it is negatively related at −0.20 with self‐reliance.

### The self‐other scale

This measures two types of social threat. First is the degree to which individuals feel they need others available and close to them in order to feel a sense of identity or even that they exist. Hoping and waiting for rescue was significantly related to this need and threat (*r* = 0.51), whereas self‐reliance negatively correlated with it (*r* = − 0.32). Importantly, and this probably links to parental overprotection, hoping and waiting for rescue was significantly related to engulfment threat at 0.35, which is the degree to which people fear that if others get close to them, they can control them.

### Mental health

Individuals scoring high on hoping and waiting for rescue also endorsed symptoms of depression, anxiety, and stress. In contrast, these variables either did not link to self‐reliance or had a negative correlation. It would seem that the feelings that one needs others to come to the rescue are associated with depression and, as noted above, lack of social safeness, and one would imagine degrees of loneliness. Although we did not measure loneliness in this study, this may be an important process, including a link with physiological effects (Cacioppo et al., 2014).

### Emotion regulation

Hoping and waiting for rescue was strongly linked to emotion regulation difficulties at 0.64, especially limited emotion regulation strategies (0.63) and impulse control difficulties (0.58). This indicates that hoping and waiting for rescue may be strongly linked to the idea that one needs others to help with emotion regulation. In attachment theory, this would be the need for others to act as a safe haven. It may be difficulties in regulating emotions that undermine people's sense of self‐efficacy and belief in themselves.

### Multiple regressions

Multiple regressions revealed that the hoping and waiting for rescue model was relatively strong, with 52% of variance explained by four predictors. Although the strongest predictor was depression, all variables were similar in their predictive power, suggesting a combination of these factors is important. The implications are that hoping and waiting for rescue is an important psychological process, with impacts on a range of other processes. Clearly, we cannot generate causal models from correlation data. Hence, we cannot say whether hoping and waiting for rescue is a vulnerability factor for depression or whether, as people become depressed, they become more passive in their coping. It is probably a two‐way street, but hoping and waiting for rescue may be a predisposing coping style to mental health difficulties, particularly as it is strongly related to emotion regulation problems. Hoping and waiting for rescue also emerged as a significant predictor in the emotion dysregulation model.

The self‐reliance model was substantially weaker, with only 14% of the variance explained by the predictors. This is important to explore in future studies with a different set of variables and possibly subgroup analyses comparing people who are self‐reliant with those who are not.

### Network analysis

The network analysis provides an overview and diagrammatic view of how the study variables relate to and correlate with each other as part of a wider network. This gives a different and wider overview of the inter‐relationship between all study variables. First, the strongest relationship was seen between early memories of warmth and safeness and maternal care. Although that in itself may not be surprising, the connection between early memories of warmth and safeness and social safeness appears to bridge the early life history variables with the cluster of mental health variables. Another finding was that social comparison and social safeness were situated closer to the mental health variables than social relating ones, suggesting they have a key role in mental well‐being. Hoping and waiting for rescue is a highly connected variable within the network, which can be seen visually in both the network and strength centrality plots. It is centered between variables of self‐identity threat, reassurance‐seeking, emotion dysregulation, and self‐reliance, suggesting its potential to influence these factors, although that will need exploring in future research.

Two questions remain. First, clearly people can be hoping and waiting for others to come and help them in an adaptive way and equally be adaptively self‐reliant. The current measure cannot clearly separate out helpful from unhelpful forms of the coping style. That is also true for self‐reliance. The scale cannot distinguish between positive forms of self‐reliance and forms that are linked to a distrust in the motives or skills of others. Interestingly, we had a specific item on social mistrust (item 4): ‘*I feel I can't trust others to be helpful’* which was removed following the factor analysis due to poor fit. However, this item has important (and significant) correlations with many study variables: maternal care (−0.29), maternal overprotection (0.18), paternal care (−0.15), paternal overprotection (0.19), early memories of warmth (−0.32), social safeness (−0.42), depression, anxiety and stress (0.27–0.36), social comparison (−0.29), reassurance‐seeking (0.23), emotion dysregulation (0.32) and engulfment threat (0.41).

Second, it is possible that ‘hoping for’ and ‘waiting for’ are subtly different processes. For example, if we are hoping for something to happen, we may get on and do other things. On the other hand, if we are waiting for something to happen and feel unable to move on without it, then that is likely to be more inhibiting. These processes need to be distinguished better than we have been able to do here.

These considerations can lead to more nuanced measures and further understanding of these complex psychological processes that can interfere with personal change.

### Clinical implications

It can be helpful for clinicians to be aware that some clients can be passive in psychotherapy and not take particularly well to self‐help or self‐practice programs, which are becoming more popular and accessible approaches. As noted by MacLeod et al. (2009), some clients struggle with motivation or sense of self efficacy, or hopelessness and helplessness when they try to engage with self‐help, even with therapist guided self‐help. Sometimes these clients can be seen as resistant or labelled as ‘not wanting to help themselves’. Our data suggest alternative explanations. One explanation is that a history of overprotection with a lack of warmth, which undermined confidence, may have left people yearning for caring support, help and connectedness. Forms of learned helplessness may also apply as an explanation. In addition, the attachment model suggests archetypal defences that switch on in certain threat contexts can inhibit explorative and self‐helping behaviour.

We suggest that therapists can actively explore this passivity and investigate potential areas of helplessness or attachment issues, where the client may have had to close down and literally wait for the carer to help. Clinicians can use socratic questions to explore the possibility that these difficulties are related to hoping and waiting for rescue issues. An opening question can be: “sometimes trying to help ourselves can seem difficult and we feel we might need others, and hope that others step in or give us the answers in some way. Do you sometimes feel like that?” From there, therapists can go on to explore the degree to which individuals hold these beliefs, how they can interfere with their own coping behaviours and explore emotional memories that they might link to. This gives clients an opportunity to explore in detail their hopes that others will somehow pick up on what they need, care for them and rescue them from their emotional distress. Indeed, some clients note that, in relationships, they rarely feel able to assertively voice their needs or desires but hope people around them will empathically pick up on them. These explorations can reveal underlying anger around childhood experiences, where they were not helped or rescued, and sometimes considerable unresolved grief and loneliness (Gilbert & Simos, 2022).

It is important that these clients are not dismissed as awkward, resistant or passive‐aggressive because they do not engage with the practices. Rather, therapists can take a step‐by‐step process of identifying the problem, offer psychoeducation on learned helplessness and separation shutdown mechanisms, and invite their exploration around those themes. This can open up feared and suppressed emotions. For one client who had much therapy and had got the label that she ‘was not motivated to help herself’, it was only through offering her a way to think about the issue, opening up unresolved attachment dynamics including anger and grief, then moving step‐by‐step in building confidence to do things for herself, that she was able to move forward.

Currently, we have attempted to identify an important psychological process that can interfere with therapeutic progress, and offer a measure that can tap into it. This then allows us to begin to think about potential therapeutic strategies to help people become more self‐reliant but also open to appropriately trusting and soliciting the help of others.

### Limitations and future directions

The present study relied on voluntary engagement from a general population sample using self‐report measures, which have their own inherent limitations. The ways in which the study concepts relate may be different in clinical populations or in groups with different adverse childhood experiences. For example, it is unclear whether a lack of early warmth would be associated with the tendency to wait for help from others, or with compulsive self‐reliance due to a mistrust of others (or a belief that others will not help) in these groups.

Participants were drawn from a UK‐based (Western) sample. Due to cross‐cultural differences in early rearing and learning experiences, for example, that some Western cultures encourage independence from an earlier age, it is unknown whether the measure will be valid in different cultures. Nonetheless, it is not anticipated that the factor structure should vary considerably, given the distinctions between items and factors.

Additionally, the first main sample and both test‐retest samples consisted of significantly more females than males. Although no gender differences were observed in the hoping and waiting for rescue and self‐reliance scales, this might not be the case with the assessment of more men and equal samples.

It is also important to understand how stable the tendencies to self‐rely or hope and wait for rescue are, and how modifiable they are when addressed by clinicians. These are key new directions for research that should be explored using longitudinal designs. Nevertheless, the scales may be useful instruments for exploring these ideas. Clearly, this is the early stage of identifying and understanding the passivity of waiting for help, but it is hoped that new measures and concepts will arise through different methodologies, including qualitative exploration.

## CONCLUSION

Our study has revealed that hoping and waiting for rescue is associated with a range of mental health and emotion regulation difficulties that may inhibit abilities to be motivated for, or engage with, self‐help practices. These are first steps in developing measures for a deeper understanding of the coping style that some clients experience: fantasies of being helped and rescued, wanting somebody to come to them rather than they themselves going out and finding help.

### AUTHOR CONTRIBUTIONS

**Paul Gilbert:** Conceptualization; scale design; visualization; supervision; writing – original draft; writing – review and editing. **Jaskaran Basran:** Project administration; data curation; visualization; writing – original draft; writing – review and editing. **Ptarmigan Plowright:** Investigation; data curation; formal analysis; visualization; writing – original draft; writing – review and editing. **Kelly Morter:** Data curation; writing – original draft; writing – review and editing. **Malcolm Schofield:** Methodology; writing – original draft; writing – review and editing. **Jean Gilbert:** Conceptualization; writing – review and editing; writing – original draft.

### CONFLICT OF INTEREST STATEMENT

The authors declare no conflict of interest.

## Supporting information


Data S1.

## Data Availability

The data that support the findings of this study are available from the corresponding author upon reasonable request.
