# Supplementary material for: Hoping and waiting for rescue: Concepts, scale development and process
Source: Psychol Psychother. 2025 Mar 28;98(3):799–820. doi: 10.1111/papt.12588 (PMC12346250; doi:10.1111/papt.12588)

**Hoping-waiting for rescue: Concepts, scale development and process**

**Supplementary Materials**

1. Weights matrix for each variable in the network analysis of sample 1.

| **Variable** | **1** | **2** | **3** | **4** | **5** | **6** | **7** | **8** | **9** | **10** | **11** | **12** | **13** | **14** | **15** | **16** |
| --- | --- | --- | --- | --- | --- | --- | --- | --- | --- | --- | --- | --- | --- | --- | --- | --- |
| HopingRescue | 0.00 | -0.20 | 0.03 | 0.00 | 0.03 | 0.00 | 0.00 | -0.08 | 0.14 | 0.00 | 0.00 | -0.09 | 0.19 | 0.14 | 0.15 | 0.04 |
| SelfReliance | -0.20 | 0.00 | -0.04 | 0.00 | -0.02 | 0.00 | 0.00 | 0.00 | 0.00 | -0.02 | 0.00 | 0.00 | -0.02 | -0.02 | -0.14 | 0.21 |
| MOTHER_Care | 0.03 | -0.04 | 0.00 | -0.13 | 0.00 | 0.00 | 0.62 | 0.00 | 0.00 | 0.00 | 0.00 | 0.00 | 0.00 | 0.00 | 0.00 | 0.00 |
| MOTHER_Overprotection | 0.00 | 0.00 | -0.13 | 0.00 | 0.00 | 0.29 | -0.01 | 0.00 | 0.00 | 0.00 | 0.04 | 0.00 | 0.10 | 0.03 | 0.00 | 0.00 |
| FATHER_Care | 0.03 | -0.02 | 0.00 | 0.00 | 0.00 | -0.12 | 0.36 | 0.01 | 0.00 | 0.00 | 0.00 | 0.00 | 0.02 | 0.00 | 0.00 | 0.00 |
| FATHER_Overprotection | 0.00 | 0.00 | 0.00 | 0.29 | -0.12 | 0.00 | -0.07 | 0.00 | 0.00 | 0.00 | 0.02 | 0.00 | 0.00 | 0.00 | 0.00 | 0.09 |
| EMWS_Total | 0.00 | 0.00 | 0.62 | -0.01 | 0.36 | -0.07 | 0.00 | 0.24 | 0.00 | 0.00 | -0.01 | 0.00 | 0.00 | 0.00 | 0.00 | 0.00 |
| SocialSafeness_Total | -0.08 | 0.00 | 0.00 | 0.00 | 0.01 | 0.00 | 0.24 | 0.00 | -0.26 | 0.00 | 0.00 | 0.32 | 0.00 | 0.00 | 0.03 | -0.16 |
| DASS_Depression | 0.14 | 0.00 | 0.00 | 0.00 | 0.00 | 0.00 | 0.00 | -0.26 | 0.00 | 0.17 | 0.20 | -0.11 | 0.00 | 0.20 | 0.00 | 0.00 |
| DASS_Anxiety | 0.00 | -0.02 | 0.00 | 0.00 | 0.00 | 0.00 | 0.00 | 0.00 | 0.17 | 0.00 | 0.41 | 0.00 | 0.12 | 0.07 | 0.00 | 0.00 |
| DASS_Stress | 0.00 | 0.00 | 0.00 | 0.04 | 0.00 | 0.02 | -0.01 | 0.00 | 0.20 | 0.41 | 0.00 | 0.00 | 0.09 | 0.22 | 0.00 | 0.21 |
| Social_Comparison | -0.09 | 0.00 | 0.00 | 0.00 | 0.00 | 0.00 | 0.00 | 0.32 | -0.11 | 0.00 | 0.00 | 0.00 | 0.00 | -0.07 | 0.00 | -0.09 |
| CORSI_Total | 0.19 | -0.02 | 0.00 | 0.10 | 0.02 | 0.00 | 0.00 | 0.00 | 0.00 | 0.12 | 0.09 | 0.00 | 0.00 | 0.22 | 0.21 | 0.00 |
| DERS_Total | 0.14 | -0.02 | 0.00 | 0.03 | 0.00 | 0.00 | 0.00 | 0.00 | 0.20 | 0.07 | 0.22 | -0.07 | 0.22 | 0.00 | 0.17 | 0.08 |
| SOS_Insecurity | 0.15 | -0.14 | 0.00 | 0.00 | 0.00 | 0.00 | 0.00 | 0.03 | 0.00 | 0.00 | 0.00 | 0.00 | 0.21 | 0.17 | 0.00 | -0.05 |
| SOS_Engulfment | 0.04 | 0.21 | 0.00 | 0.00 | 0.00 | 0.09 | 0.00 | -0.16 | 0.00 | 0.00 | 0.21 | -0.09 | 0.00 | 0.08 | -0.05 | 0.00 |

1. Weights matrix for each variable in the network analysis of sample 2

| **Variable** | **1** | **2** | **3** | **4** | **5** | **6** | **7** | **8** | **9** | **10** | **11** | **12** | **13** | **14** | **15** | **16** |
| --- | --- | --- | --- | --- | --- | --- | --- | --- | --- | --- | --- | --- | --- | --- | --- | --- |
| HopingRescue | 0.00 | -0.20 | -0.02 | 0.00 | 0.00 | 0.10 | 0.00 | -0.03 | 0.10 | 0.00 | 0.00 | 0.00 | 0.14 | 0.28 | 0.21 | 0.03 |
| SelfReliance | -0.20 | 0.00 | -0.03 | 0.00 | 0.00 | 0.00 | 0.00 | 0.00 | 0.00 | 0.00 | 0.00 | 0.00 | 0.00 | 0.00 | -0.16 | 0.19 |
| MOTHER_Care | -0.02 | -0.03 | 0.00 | -0.20 | 0.03 | 0.00 | 0.55 | 0.00 | 0.00 | 0.04 | 0.00 | 0.01 | 0.00 | -0.02 | 0.00 | 0.00 |
| MOTHER_Overprotection | 0.00 | 0.00 | -0.20 | 0.00 | 0.00 | 0.25 | -0.07 | 0.00 | 0.01 | 0.00 | 0.00 | 0.00 | 0.03 | 0.03 | 0.00 | 0.07 |
| FATHER_Care | 0.00 | 0.00 | 0.03 | 0.00 | 0.00 | -0.02 | 0.33 | 0.01 | 0.00 | 0.00 | 0.00 | 0.06 | 0.00 | 0.00 | 0.00 | 0.00 |
| FATHER_Overprotection | 0.10 | 0.00 | 0.00 | 0.25 | -0.02 | 0.00 | -0.06 | 0.00 | 0.00 | 0.01 | 0.00 | 0.00 | 0.05 | 0.00 | 0.00 | 0.01 |
| EMWS_Total | 0.00 | 0.00 | 0.55 | -0.07 | 0.33 | -0.06 | 0.00 | 0.27 | 0.00 | 0.00 | 0.00 | 0.02 | 0.00 | -0.07 | 0.00 | -0.10 |
| SocialSafeness_Total | -0.03 | 0.00 | 0.00 | 0.00 | 0.01 | 0.00 | 0.27 | 0.00 | -0.30 | 0.00 | 0.00 | 0.36 | 0.00 | 0.00 | 0.11 | -0.02 |
| DASS_Depression | 0.10 | 0.00 | 0.00 | 0.01 | 0.00 | 0.00 | 0.00 | -0.30 | 0.00 | 0.16 | 0.29 | -0.08 | 0.00 | 0.07 | 0.00 | 0.05 |
| DASS_Anxiety | 0.00 | 0.00 | 0.04 | 0.00 | 0.00 | 0.01 | 0.00 | 0.00 | 0.16 | 0.00 | 0.41 | 0.00 | 0.08 | 0.17 | 0.00 | 0.00 |
| DASS_Stress | 0.00 | 0.00 | 0.00 | 0.00 | 0.00 | 0.00 | 0.00 | 0.00 | 0.29 | 0.41 | 0.00 | 0.00 | 0.08 | 0.23 | 0.02 | 0.08 |
| Social_Comparison | 0.00 | 0.00 | 0.01 | 0.00 | 0.06 | 0.00 | 0.02 | 0.36 | -0.08 | 0.00 | 0.00 | 0.00 | 0.00 | -0.16 | 0.00 | 0.00 |
| CORSI_Total | 0.14 | 0.00 | 0.00 | 0.03 | 0.00 | 0.05 | 0.00 | 0.00 | 0.00 | 0.08 | 0.08 | 0.00 | 0.00 | 0.15 | 0.36 | 0.15 |
| DERS_Total | 0.28 | 0.00 | -0.02 | 0.03 | 0.00 | 0.00 | -0.07 | 0.00 | 0.07 | 0.17 | 0.23 | -0.16 | 0.15 | 0.00 | 0.01 | 0.08 |
| SOS_Insecurity | 0.21 | -0.16 | 0.00 | 0.00 | 0.00 | 0.00 | 0.00 | 0.11 | 0.00 | 0.00 | 0.02 | 0.00 | 0.36 | 0.01 | 0.00 | 0.00 |
| SOS_Engulfment | 0.03 | 0.19 | 0.00 | 0.07 | 0.00 | 0.01 | -0.10 | -0.02 | 0.05 | 0.00 | 0.08 | 0.00 | 0.15 | 0.08 | 0.00 | 0.00 |

1. Normalized centrality statistics for each variable, for each sample.

|  | Sample 1 | | | | Sample 2 | | | |
| --- | --- | --- | --- | --- | --- | --- | --- | --- |
|  | Betweenness | Closeness | Strength | Expected influence | Betweenness | Closeness | Strength | Expected influence |
| Hoping for rescue | -0.43 | -0.08 | 0.77 | -0.28 | 0.64 | 0.45 | 0.69 | 0.27 |
| Self-reliance | -0.69 | -0.56 | -0.97 | -1.69 | -0.87 | -0.93 | -1.13 | -2.07 |
| Maternal care | -0.52 | -0.35 | -0.31 | 0.02 | 0.11 | 0.20 | -0.01 | -0.45 |
| Maternal overprotection | -0.35 | -1.25 | -1.18 | -0.36 | -0.34 | -1.40 | -0.85 | -1.11 |
| Paternal care | -0.52 | -0.97 | -1.37 | -0.47 | -0.96 | -0.77 | -1.58 | -0.30 |
| Paternal overprotection | -0.60 | -1.56 | -1.25 | -0.63 | -0.52 | -1.64 | -1.43 | -0.53 |
| Early memories of warmth and safeness | 1.55 | 0.39 | 1.69 | 1.58 | 2.15 | 1.16 | 1.96 | 1.01 |
| Social safeness and pleasure | 2.50 | 1.86 | 0.82 | -0.88 | 2.24 | 1.88 | 0.69 | -0.32 |
| Depression | 1.47 | 1.52 | 0.72 | -0.32 | 0.11 | 0.84 | 0.61 | -0.60 |
| Anxiety | -0.86 | -0.14 | -0.42 | 0.68 | -0.96 | -0.64 | -0.11 | 1.01 |
| Stress | -0.17 | 0.63 | 1.19 | 1.66 | 0.02 | 0.65 | 0.73 | 1.68 |
| Difficulties in emotion regulation (DERS) | -0.86 | -0.30 | -0.87 | -1.21 | -0.25 | 0.52 | -0.77 | -0.92 |
| Social comparison | -0.17 | -0.16 | 0.33 | 1.12 | -0.87 | -0.64 | 0.49 | 1.47 |
| Reassurance-seeking (CORSI) | 0.43 | 0.88 | 1.30 | 1.36 | 0.64 | 1.02 | 1.29 | 0.73 |
| Insecurity threat to self-construction (SOS) | -0.86 | -1.06 | -0.59 | -0.25 | -0.43 | 0.05 | -0.09 | 0.12 |
| Engulfment threat to self-construction (SOS) | 0.09 | 1.14 | 0.13 | -0.33 | -0.70 | -0.74 | -0.50 | 0.02 |

1. Centrality stability plot using 1000 case-drop bootstrap samples with an increasing number of dropped cases. Shaded areas indicate confidence intervals.


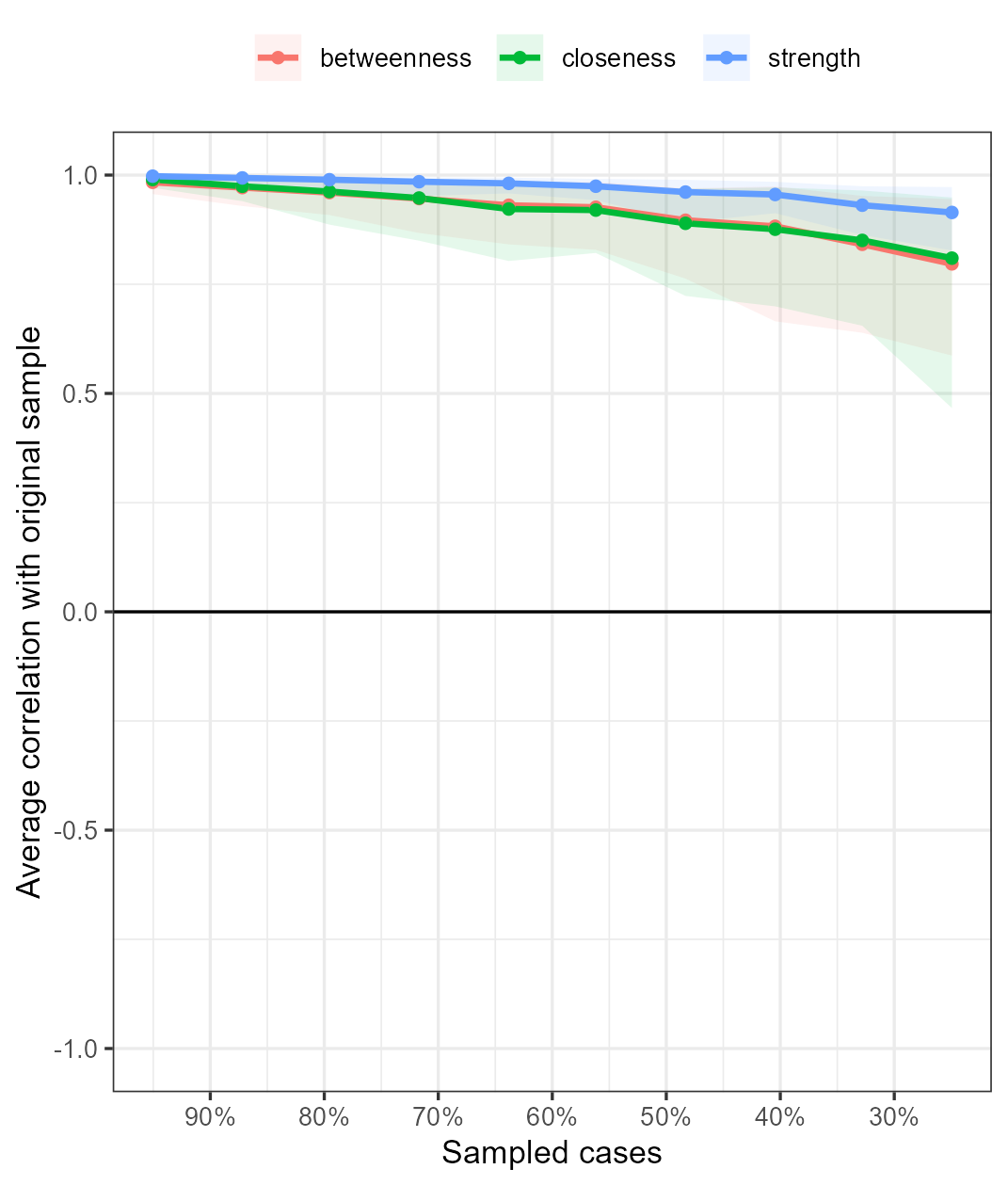


1. Edge stability plot using 1000 nonparametric bootstrap samples. The *y*-axis shows all edges within the network (e.g. depression-emotion dysregulation). Edge labels have been omitted for clarity. Edge weights are shown on the *x*-axis. The shaded grey area denotes 95% confidence intervals.


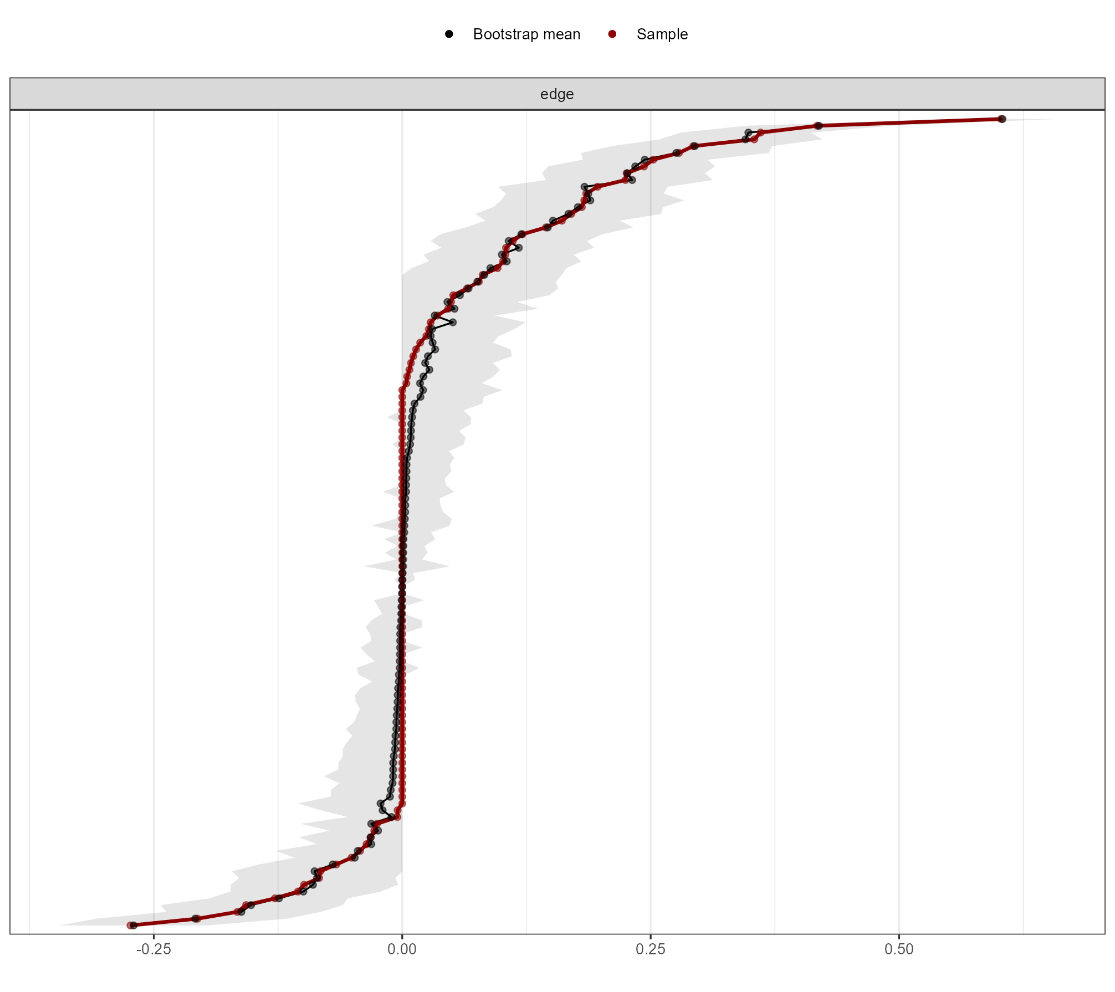

Supplement: Supplementary file 1 — Data S1. [file PAPT-98-799-s001.docx]
